# It Takes Two to Tango: Customization and Standardization as Colluding Logics in Healthcare: Comment on "(Re) Making the Procrustean Bed Standardization and Customization as Competing Logics in Healthcare"

**DOI:** 10.15171/ijhpm.2017.77

**Published:** 2017-06-28

**Authors:** David Greenfield, Kathy Eljiz, Kerryn Butler-Henderson

**Affiliations:** Australian Institute of Health Service Management, University of Tasmania, Sydney, Australia.

**Keywords:** Healthcare, Standardization, Customization

## Abstract

The healthcare context is characterized with new developments, technologies, ideas and expectations that are continually reshaping the frontline of care delivery. Mannion and Exworthy identify two key factors driving this complexity, ‘standardization’ and ‘customization,’ and their apparent resulting paradox to be negotiated by healthcare professionals, managers and policy makers. However, while they present a compelling argument an alternative viewpoint exists. An analysis is presented that shows instead of being ‘competing’ logics in healthcare, standardization and customization are long standing ‘colluding’ logics. Mannion and Exworthy’s call for further sustained work to understand this complex, contested space is endorsed, noting that it is critical to inform future debates and service decisions.

## Introduction

The evolving and complex healthcare sector presents continual change opportunities and challenges for professionals and the organizations in which they work.^1^ Two key factors driving this complexity are articulated succinctly by Mannion and Exworthy.^2^ They have described the factors of ‘standardization’ and ‘customization,’ and the apparent paradox to be negotiated by healthcare professionals, managers and policy makers.^2^ In healthcare, standardizing is the enactment of care activities designed to promote uniformity, stability and commensurability of professional thought, meaning, actions and outcomes. While customization is ‘co-producing’ care through tailoring processes and services to suit the understanding, beliefs, wishes and needs of the patient and their family. However, while Mannion and Exworthy present a compelling argument an alternative perspective comes to mind. This is, standardization and customization are long standing ‘colluding,’ not recent ‘competing,’ logics in healthcare.

## The Collusion of Standardization and Customization

Healthcare, in the relatively recent past of the early-to-mid twentieth century, was a cottage industry.^3^ Healthcare practices were driven by local educational networks and the knowledge within them. Where and with whom an individual was educated and interned defined the basis for their clinical work. A characteristic of human behavior, in any endeavor, is to simplify and standardize how we think and do things.^4^ In healthcare this has been no different and clinicians learnt behavior in this way has been termed ‘mindlines.’^5^ Individuals learnt and adopted simplified, standardized ways of delivering care, for example, the taking of a medical history, use of a rounding process on hospital wards or treatment of a particular condition, and adapted these to each individual patient or organizational setting.^6^ Patients deferred to professional judgement, at least explicitly. There was little professional oversight and an absence of external professional regulation. For the first two-thirds of the twentieth century healthcare was, by our current expectations, basic physical care and largely un-technological. The diagnostic tests and technical instruments available, while increasing in ability and number, were limited and where used, straightforward and consistent in their application.^7^ There was not scope for significant variability nor to alter the options for individual patients; standardization was customization, and customization was standardization. Clinical practices were localized standardized behaviors that were customized with patients by individual clinicians.^3^ In this context, standardization and customization were intertwined elements of elementary healthcare practice.

Within a period of only 50 years, however, we have witnessed extraordinary changes in healthcare. There has been a fast evolution of knowledge, technology and expectations. Technological, scientific, industry, economic, educational and managerial developments, from a host of other sectors, have flowed through to enable significant, ongoing changes in healthcare.^8^ The health industry, as a result, has been lifted out from the cottage industry of the early twentieth century by the industrialization of medicine. Healthcare, now, is a large scale, economically vital, diverse, multi-professional, technological, knowledge and expertize based industry servicing whole populations.^1,8^ These changes have delivered large scale complex organizations – tertiary teaching hospitals - with an extraordinary diversity of services. These hospitals are characterized by complex of interactions between people, systems and technologies. Increasing specialization within professions, and further standardization and customization of services and organizations has been a result, and, in turn, become more possible because of these developments. New technologies, data^9^ and knowledge enable, and necessitate, that services and care are progressively specialized. Within new sub-specialties the micro-elements of care are increasingly standardized and customized in ways previously not possible.

Medicine, nursing and allied health professions and services are now more accurately and meaningfully described by their respective sub-specialties within organizational settings^10^; for example, emergency care physician or nurse, as distinct from an intensive care physician or nurse.^11^ In this way, as roles and practices within specific settings become specialized they also become governed by tighter sets of norms, rules and behaviors - about who practices in which space, and what they can and cannot do.^10,12^ The tighter governance has been driven, in part, by the need to improve safety and quality because of the diverse technologies, data and knowledge involved.^9,13^ Inappropriate healthcare is inefficient, unsustainable and at times can, and does, kill rather than cure.^14^ Improved governance has been achieved by professions internally^3^ and the State externally^1^ imposing standards and regulations.^13^ An inherent challenge to achieving safety and quality improvements is shifting individual clinicians focus from individual patients to consider organisational and system issues.^3^ Clinicians are increasingly expected to engage with individual patient and system outcomes. Within this context, empirical studies, drawing on sociological theories, have revealed how the professional and patient interact to define and produce a set of healthcare practices, particular to a service and space.^15^ In doing so, the role, actions and expectations of the patient are recognized as equally important as that of the professional. In the modern context as ‘calculating selves’ ‘patients’ became ‘customers’ and assumed, willingly or not, responsibility for their own health and treatment decisions.^15^ The new ‘standardization’ in health is one where there is explicit shared decision making between patients and health professionals to ‘customize’ care for each individual. With this perspective, standardization and customization are not divergent and opposing, nor are they newly introduced elements. They are long-standing, colluding and enabling elements; one begets the other and reinforces their effects. Or, to put it another way, “it takes two to tango.”

## Accountability and Fault-Lines Across Professionals and Patients

The health system began, evolved and now actively promotes and builds upon, standardization and customization; a point acknowledged by Mannion and Exworthy.^2^ Together they collude to hold both health professionals and patients ‘accountable’ for their expectations, thoughts and actions. However, there are incongruences that arise. Within the framework of the industrialization of medicine, our modern healthcare system is identified as being at the second stage: standardization.^2^ Health professionals being held accountable for their practice, rather than the role of patients, is normally the primary focus. Standardizing, as noted, is the enactment of activities designed to promote uniformity, stability and commensurability of professional thought, meaning, actions and outcomes. However, as has been well reported, including by Mannion and Exworthy,^2^ standardization can *promote* but does not *ensure* uniformity of healthcare. Particularly in clinical arenas, there is significant resistance to standardization viewing it as a threat to autonomy and denial of professional expertise. As noted, standardization has always been an element of health professional practice, that is, the requirement to learn, master and display the appropriate knowledge, skills and behaviors of a chosen discipline. Resistance emerges when others, sometimes from within the profession and at other times external to it, seek to direct practice and the individual perceives a loss of control. However, if standardization applies to professionals does it not apply equally to patients?

Customization is explained as being enacted through four components, that is, of: systems; services and treatments; individual choice; and personal responsibility. Here, in contrast, individual patients, and/or their carers, are focused on and sought to be held accountable for their health and actions, more so than professionals. Similarly to clinical professionals resisting standardization, individual patients capacity to engage with and fulfil the ‘customer’ role in a healthcare setting is fraught and contested in many circumstances. But, in reverse, if customization applies to patients does it not apply equally to professionals?

From this perspective, the framing of ‘resistance’ for health professionals and patients is instructive. For health professionals the notion is presented in terms positively highlighting their knowledge and independence – “undermining expertise and autonomy”; by contrast, for patients the description is in phrasing emphasising negative thoughts and emotions – “… increased obligations and expectations … guilt and anxiety if they do not fulfil the expectations.” Hence, therein the answer to our two questions lies and a critical fault-line in healthcare emerges. A fracture occurs through the inconsistent and contested application and translation of standardization and customization requirements for professionals and patients. An outcome being, that we are dealing with a wicked problem,^16^ which by definition, is of our own making. To deal with this situation requires collaboration between professionals, and with patients, the use of systems thinking, as well as the development and implementation of multiple change strategies.

## What Does the Future Hold?

So, what does this mean for the direction of healthcare and accompanying future research agenda? Applying the notion of ‘institutional logics’^17,18^ to investigate where we are and where we are heading is, as Mannion and Exworthy^2^ suggest, a promising idea. The contribution of other logics – such as professionalism and managerialism^19^ – and theoretical frameworks – for example, local universality^20^ – also have the potential to illuminate expectations, practices and professional-patient relationships, and challenge thinking and implications. These different ideas will enable us to address the critical questions facing us: how will the frontline of healthcare delivery continue to be conceptualized, described and provided? Is the frontline now – if we are ‘calculating selves’ – what individuals expect and do for themselves in their own home? What will be the intertwined roles of professionals and patients-customers? What will be gained as much as what will be lost? Exploring and understanding what will arise from the ongoing standardization-customization collusion, explicitly and implicitly, and for whom and how services will be provided, is critical to inform ongoing debates and future decisions.

## Ethical issues

Not applicable.

## Competing interests

Authors declare that they have no competing interests.

## Authors’ contributions

All authors contributed to the writing of and approved the final manuscript.
